# Machine-learning assisted modelling of multiple elements for authenticating edible animal blood food

**DOI:** 10.1016/j.fochx.2022.100280

**Published:** 2022-03-07

**Authors:** Fangkai Han, Joshua H. Aheto, Marwan M.A. Rashed, Xingtao Zhang

**Affiliations:** a School of Biological and Food Engineering, Suzhou University, Bianhe Middle Road 49, Suzhou 234000, Anhui, PR China; b School of Food and Biological Engineering, Jiangsu University, Xuefu Road 301, Zhenjiang 212013, Jiangsu, PR China

**Keywords:** Animal blood food, Multi-element analysis, ICP-MS, Machine learning, Blood tofu

## Abstract

Elemental fingerprint coupled with machine learning modelling was proposed for species authentication of the edible animal blood gel (EABG). A total of 25 elements were determined by inductively coupled plasma mass spectrometry (ICP-MS) and atomic absorption spectroscopy (AAS) in 150 EABG samples prepared from five species of animals, namely duck, chicken, bovine, pig, and sheep. Extreme learning machine (ELM) models were constructed and optimized. Principal component analysis and Fisher linear discriminant analysis were comparatively utilized for dimension reduction of the crucial input elements selected via stepwise discriminant analysis and one-way ANOVA. The optimal ELM model was obtained with the crucial elements selected by one-way ANOVA from the relative content of the measured elements, which afforded accuracies of 98.0% and 96.0% for the training and test set, respectively. All findings suggest that elemental fingerprint accompanied by ELM have great potential in authenticating the edible animal blood food.

## Highlights

• The critical elements for identifying species of the animal blood food were selected.
• Elemental fingerprint coupled with ELM were proposed for species identification of the animal blood food.
• The optimal ELM model for identifying the species of the animal blood food was constructed.
• The absolute and relative content of 25 elements in animal blood food were reported for the first time.

## Introduction

Animal blood is rich in protein with high biological value, so it is accepted as value-added component of foods or dietary supplements in many societies (Bah et al., 2013, Toldrá et al., 2012). The high economic animal blood food, known as “blood tofu” usually made via simple heating of the fresh edible animal blood is widely distributed in the supermarkets or restaurants of China. Generally, blood from duck, pig and chicken are used for preparing blood tofu. However, in view of its unique taste and texture, duck blood tofu is most popular in China. Therefore, the price of duck blood tofu is usually higher than that of other animal blood tofu, which contributes to food fraud, adulteration, and mislabeling.

Currently, the high economic value of duck blood tofu adulterated or replaced with other low-price animal blood is still a serious social problem implied by media reports and administrative punishment cases (Zhang, Wang, Ma, Li, & Li, 2020). Hence, a method for authentication of animal blood foods is needed. Specific biomarkers, such as DNA (Cheng et al., 2014, El-Sayed et al., 2010, Unajak et al., 2010) and peptide (Zhang et al., 2020), have shown great potential for the aforementioned purpose. However, although the specific biomarker-based analytical techniques are accurate, these techniques can be practically problematic due to sample contamination and intentional addition/removal of the biomarkers by the food counterfeiters. Therefore, it is urgent to develop a novel technology to authenticate animal blood foods.

Elemental fingerprint showed a unique advantage to the specific biomarkers-based analytical techniques as it involves all chemical constituents in foods making it difficult for the food counterfeiters to adjust such huge element species and content. Recently, the elemental fingerprint has been used to trace the origin of green coffee beans (Endaye et al., 2020), determine the geographic origin of salmonid (Han, Dong, Li, Wei, Zhou, & Gao, 2020), discriminate geographical origin and species of China’s cattle bones (Zhang et al., 2021), and authenticate the geographical origin of Australian Cabernet Sauvignon wines (Ranaweera, Gilmore, Capone, Bastian, & Jeffery, 2021), to name but a few. Nevertheless, to the best of the authors’ knowledge, no studies investigating the authentication of animal blood foods based on elemental fingerprints have been reported. Therefore, the present work aims to develop a novel technique for species authentication of the edible animal blood gel (EABG) using elemental fingerprint coupled with machine learning modelling.

## Materials and methods

### Samples preparation

Fresh animal blood samples were collected in Suzhou, China from July 20th to August 7th, 2021. Duck and chicken blood samples were purchased from the vendor for on-site slaughter at a local farmers' market; a local slaughterhouse provided pig and bovine blood samples; the sheep blood samples were purchased from a local hotpot restaurant. All the fresh animal blood samples were transported to the laboratory in an ice-filled box and then sterilized under high temperature at 121 °C for 30 min after natural sedimentation. All blood samples were homogenized separately, and then frozen at −20 °C. Eventually, thirty blood samples for each species of the animals were collected, thus yielding a total of 150 EABG samples for multi-element measurements.

### Multi-element analysis of the EABG

According to Chinese standard GB 5009.268–2016 (National Health and Family Planning Commission of the People’s Republic of China, 2016a), trace elements such as lithium (Li), beryllium (Be), boron (B), aluminum (Al), titanium (Ti), vanadium (V), chromium (Cr), manganese (Mn), iron (Fe), cobalt (Co), nickel (Ni), copper (Cu), zinc (Zn), arsenic (As), selenium (Se), rubidium (Rb), strontium (Sr), cadmium (Cd), barium (Ba), thallium (Tl), and lead (Pb) were determined by using inductively coupled plasma mass spectrometry (ICP-MS). The atomic absorption spectroscopy (AAS) was utilized for the macroelement measurements, potassium (K) and sodium (Na) were tested according to GB 5009.91–2017 (National Health and Family Planning Commission of the People’s Republic of China, 2017a), calcium (Ca) and magnesium (Mg) were analyzed according to GB 5009.92–2016 (National Health and Family Planning Commission of the People’s Republic of China, 2016b) and GB 5009.241–2017 (National Health and Family Planning Commission of the People’s Republic of China, 2017b), respectively. All elemental analyses were carried out according to Chinese national standards, which is convenient for potential users to follow.

### Chemometrics and software

Herein, the absolute and relative contents of the measured elements were used as the original dataset for machine learning modelling. Because of the big differences in contents of the microelements and macro-elements, Z-score normalization was performed firstly on the original datasets to eliminate the data orders. Then, the stepwise discriminant analysis (SWDA) was compared with one-way analysis of variance (ANOVA) for selecting crucial elements; principal component analysis (PCA) and Fisher linear discriminant analysis (Fisher LDA) were implemented comparatively to reduce dimension; eventually, extreme learning machine (ELM) was selected for modeling due to its simple network structure, good generalization ability and less time consuming (Han et al., 2020, Huang et al., 2006).

During ELM modelling, the number of hidden neurons and the activation function of the hidden layers were optimized. As the strategy of cut-and-trial was used, the optimal number of hidden neurons was set at a range of [1, 100]. Also, the frequently used activation functions for the hidden layers are depicted in the following formulas:.

(1) $$\mathrm{Sig}:S\left(x\right)=\frac{1}{1+e^{-x}}$$

(2) Sin:S(x)=sin(x)

(3) $$\mathrm{Hard}\lim:S\left(x\right)=\left(\frac{1\chi >0}{0x<0}\right)$$

where: x means the inputs of these formulas.

Performance of the constructed ELM model was evaluated by using the recognition accuracy, which is calculated by dividing the number of correctly predicted samples by the total number of samples in the training or test set. All algorithms in this work were implemented with Windows 10 in Matlab version 7.14 (Mathworks, Natick, USA).

## Results and discussions

### Elements content in different species of EABG

Results of the elemental analysis for the EABG samples are shown in Table 1. According to the results, there was no significant difference in the contents of Li, Be, Ti, Co, As, Cd, Tl, and Pb in EABG from different species of animals. Elements were different between only two species of EABG, but no significant differences with other remaining three species of EABG were B, Al, Mn, and Na, and more details are shown as follows: B content in bovine blood gel was significantly higher than that in sheep blood gel, but similar with that in blood gels made from duck, chicken, and pig; Al content in pig blood gel was significantly higher than that in bovine blood gel, but similar with that in duck, chicken, and sheep blood gels; Mn content in chicken blood gel was found significantly higher than that in bovine blood gel, but similar with that in duck, pig, and sheep blood gels; Na content in bovine blood gel was significantly higher than that in pig blood gel, but similar with that in duck, chicken, and sheep blood gels.

**Table 1:** The absolute content of the elements measured in blood gels prepared from duck, chicken, bovine, pig, and sheep.

	Duck (mg/kg)	Chicken (mg/kg)	Bovine (mg/kg)	Pig (mg/kg)	Sheep (mg/kg)
Li	0.0473 ± 0.0252^a^	0.043 ± 0.0191^a^	0.0418 ± 0.0312^a^	0.0357 ± 0.0195^a^	0.0426 ± 0.0137^a^
Be	0.0798 ± 0.1812^a^	0.1876 ± 0.3958^a^	0.236 ± 0.5341^a^	0.0946 ± 0.2598^a^	0.0892 ± 0.2512^a^
B	0.275 ± 0.2226^ab^	0.221 ± 0.163^ab^	0.537 ± 1.37^a^	0.284 ± 0.301^ab^	0.203 ± 0.130^b^
Al	1.62 ± 0.922^ab^	2.17 ± 2.98^ab^	1.42 ± 1.07^b^	2.62 ± 1.07^a^	2.19 ± 3.18^ab^
Ti	0.593 ± 0.522^a^	0.593 ± 0.619^a^	0.818 ± 1.25^a^	0.579 ± 0.680^a^	0.523 ± 0.469^a^
V	0.0493 ± 0.0943^ab^	0.0795 ± 0.166^ab^	0.142 ± 0.341^a^	0.0483 ± 0.118^ab^	0.0401 ± 0.116^b^
Cr	0.154 ± 0.182^b^	0.285 ± 0.163^ab^	0.385 ± 0.412^a^	0.283 ± 0.133^ab^	0.244 ± 0.138^b^
Mn	0.158 ± 0.147^ab^	0.209 ± 0.154^a^	0.119 ± 0.129^b^	0.174 ± 0.0712^ab^	0.181 ± 0.0983^ab^
Fe	809 ± 129^c^	5709 ± 152^a^	728 ± 280^c^	948 ± 134^b^	730 ± 117^c^
Co	0.0200 ± 0.0181^a^	0.0153 ± 0.0351^a^	0.0164 ± 0.0500^a^	0.00740 ± 0.0172^a^	0.00720 ± 0.0182^a^
Ni	0.0855 ± 0.104^b^	0.287 ± 0.637^a^	0.157 ± 0.0617^ab^	0.179 ± 0.0939^ab^	0.134 ± 0.0412^b^
Cu	0.597 ± 0.314^c^	0.908 ± 1.74^c^	2.43 ± 0.532^a^	1.50 ± 0.164^b^	1.44 ± 0.228^b^
Zn	6.18 ± 3.96^b^	8.416 ± 8.10^b^	119 ± 440^a^	10.4 ± 19.2^b^	3.89 ± 0.725^b^
As	0.00790 ± 0.00680^a^	0.00750 ± 0.00600^a^	0.00990 ± 0.0156^a^	0.00650 ± 0.00430^a^	0.00590 ± 0.00400^a^
Se	0.327 ± 0.0670^a^	0.244 ± 0.0552^c^	0.312 ± 0.0812^ab^	0.345 ± 0.0795^a^	0.286 ± 0.0529^b^
Rb	0.0530 ± 0.0294^ab^	0.0394 ± 0.0158^b^	0.0505 ± 0.0288^ab^	0.0639 ± 0.0413^a^	0.0426 ± 0.0185^b^
Sr	0.144 ± 0.0725^b^	0.159 ± 0.0767^b^	0.246 ± 0.109^a^	0.148 ± 0.0424^b^	0.232 ± 0.0744^a^
Cd	0.0134 ± 0.0321^a^	0.0349 ± 0.0835^a^	0.0283 ± 0.0905^a^	0.00680 ± 0.0248^a^	0.00980 ± 0.0282^a^
Ba	0.101 ± 0.0423^b^	0.094 ± 0.0641^b^	0.144 ± 0.0948^a^	0.0902 ± 0.0355^b^	0.142 ± 0.0543^a^
Tl	0.0841 ± 0.258^a^	0.300 ± 0.775^a^	0.244 ± 0.685^a^	0.0992 ± 0.337^a^	0.104 ± 0.353^a^
Pb	0.318 ± 0.163^a^	0.391 ± 0.707^a^	0.308 ± 0.527^a^	0.243 ± 0.165^a^	0.270 ± 0.229^a^
Na	1880 ± 401^ab^	1764 ± 293^ab^	2050 ± 400^a^	1500 ± 1850^b^	1840 ± 517^ab^
Mg	148 ± 27.7^a^	86.5 ± 13.5^b^	46.7 ± 6.97^b^	153 ± 207^a^	50.2 ± 10.7^b^
Ca	90.1 ± 39.1^a^	101 ± 25.9^a^	96.2 ± 18.1^a^	60.3 ± 81.8^b^	59.0 ± 41.1^b^
K	1760 ± 388^b^	1730 ± 300^b^	646 ± 368^c^	2300 ± 2200^a^	1030 ± 124^c^

Results are expressed as mean values ± standard deviation, n = 30. Values in the same line with different superscripts were significantly different (*P* < 0.05).

As demonstrated in Table 1, elements content in two species of blood gels showed significant differences with other remaining three species animal blood gels were Sr, Ba, Mg, and Ca and more details are shown as follows: Sr content in bovine and sheep blood gels were significantly higher than that in duck, chicken, and pig blood gels; Ba content in bovine and sheep blood gels were also significantly higher than that in duck, chicken, and pig blood gels; Mg content in duck and pig blood gels was significantly higher than that in chicken, bovine, and beef blood gels; Ca content in pig and sheep blood gels was significantly higher than that in duck, chicken, and bovine blood gels.

The Table 1 also shows that Fe content in the EABG samples increased in the following order: chicken, pig and duck; no significant difference was found in Fe content in EABG samples of duck, cow and sheep. Cu content increased in the following order: bovine, pig and duck; it was found that the Cu content was similar between pig and sheep blood gels, and between duck and chicken blood gels. K content found in the EABG samples increased in the following order: pig, duck and bovine; it was found that the K content was similar between duck and chicken blood gels, and between bovine and sheep blood gels. Zn content in bovine blood gel was significantly higher than in other animal blood gels used. V content in bovine blood gel was significantly higher than that in sheep blood gel, but there were no significant differences with other remaining animal blood gels. Cr content in bovine blood gel was significantly higher than that in duck and sheep blood gels, but there was no significant difference with chicken and pig blood gels. Se content found in the EABG samples increased in the following order: pig, sheep and chicken; it was found that the Se content was similar between duck and pig blood gels as well as between sheep and bovine blood gels.

In order to further explore the difference of EABG multi-element distribution, the relative content of the measured elements obtained via single element content divided by the total element content of the sample was also analyzed, and the results are shown in Table 2.

**Table 2:** Results of the relative content of elements in blood gels (duck, chicken, bovine, pig, and sheep) obtained via single element content divided by the total element content of the sample.

	Duck (%)	Chicken (%)	Bovine (%)	Pig (%)	Sheep (%)
Li	1.02*10^-5^ ± 0.565*10^-5 ab^	1.02*10^-5^ ± 0.449*10^-5 ab^	1.16*10^-5^ ± 0.948*10^-5 a^	0.836*10^-5^ ± 0.496*10^-5b^	1.17*10^-5^ ± 0.404*10^-5 a^
Be	1.68*10^-5^ ± 3.65*10^-5 a^	4.47*10^-5^ ± 9.53*10^-5 a^	5.59*10^-5^ ± 11.0*10^-5 a^	2.17*10^-5^ ± 6.01*10^-5 a^	2.60*10^-5^ ± 7.42*10^-5 a^
B	5.99*10^-5^ ± 5.06*10^-5 a^	5.17*10^-5^ ± 3.66*10^-5 a^	10.4*10^-5^ ± 19.5*10^-5 a^	6.63*10^-5^ ± 7.11*10^-5 a^	5.54*10^-5^ ± 3.94*10^-5 a^
Al	3.54*10^-4^ ± 2.18*10^-4c^	5.07*10^-4^ ± 7.01*10^-4 abc^	3.89*10^-4^ ± 2.91*10^-4 bc^	6.06*10^-4^ ± 2.77*10^-4 ab^	6.15*10^-4^ ± 9.26*10^-4 a^
Ti	1.27*10^-4^ ± 1.11*10^-4 a^	1.39*10^-4^ ± 1.45*10^-4 a^	2.10*10^-4^ ± 2.98*10^-4 a^	1.33*10^-4^ ± 1.49*10^-4 a^	1.47*10^-4^ ± 1.39*10^-4 a^
V	1.04*10^-5^ ± 1.89*10^-5b^	1.89*10^-5^ ± 4.00*10^-5 ab^	3.27*10^-5^ ± 6.31*10^-5 a^	1.11*10^-5^ ± 2.72*10^-5b^	1.16*10^-5^ ± 3.42*10^-5b^
Cr	3.21*10^-5^ ± 3.64*10^-5c^	6.77*10^-5^ ± 4.02*10^-5b^	9.91*10^-5^ ± 6.56*10^-5 a^	6.57*10^-5^ ± 3.30*10^-5b^	6.83*10^-5^ ± 4.37*10^-5b^
Mn	3.42*10^-5^ ± 3.32*10^-5b^	4.96*10^-5^ ± 3.71*10^-5 a^	3.04*10^-5^ ± 2.39*10^-5b^	3.93*10^-5^ ± 1.54*10^-5 ab^	4.97*10^-5^ ± 2.86*10^-5 a^
Fe	0.173 ± 0.0252 ^a^	0.134 ± 0.0337 ^d^	0.197 ± 0.0460^b^	0.221 ± 0.0504 ^a^	0.198 ± 0.0354^b^
Co	4.29*10^-6^ ± 3.84*10^-6 a^	3.65*10^-6^ ± 8.51*10^-6 a^	3.21*10^-6^ ± 6.83*10^-6 a^	1.70*10^-6^ ± 4.00*10^-6 a^	2.06*10^-6^ ± 5.38*10^-6 a^
Ni	1.81*10^-5^ ± 2.18*10^-5b^	6.31*10^-5^ ± 12.5*10^-5 a^	4.38*10^-5^ ± 1.84*10^-5 ab^	4.22*10^-5^ ± 2.53*10^-5 ab^	3.71*10^-5^ ± 1.44*10^-5 ab^
Cu	1.26*10^-4^ ± 5.65*10^-5c^	20.2*10^-5^ ± 34.0*10^-5c^	68.9*10^-5^ ± 20.4*10^-5 a^	35.2*10^-5^ ± 7.78*10^-5b^	39.4*10^-5^ ± 8.80*10^-5b^
Zn	1.36*10^-3^ ± 1.05*10^-3b^	1.94*10^-3^ ± 1.78*10^-3b^	17.2*10^-3^ ± 60.0*10^-3 a^	2.42*10^-3^ ± 4.45*10^-3b^	1.06*10^-3^ ± 0.235*10^-3b^
As	1.69*10^-6^ ± 1.39*10^-6 ab^	1.77*10^-6^ ± 1.43*10^-6 ab^	2.55*10^-6^ ± 3.67*10^-6 a^	1.48*10^-6^ ± 1.01*10^-6b^	1.64*10^-6^ ± 1.18*10^-6 ab^
Se	7.00*10^-5^ ± 1.40*10^-5b^	5.79*10^-5^ ± 1.41*10^-5c^	8.82*10^-5^ ± 2.95*10^-5 a^	8.02*10^-5^ ± 2.36*10^-5 ab^	7.84*10^-5^ ± 1.79*10^-5 ab^
Rb	1.14*10^-5^ ± 0.618*10^-5 ab^	0.933*10^-5^ ± 0.385*10^-5b^	1.43*10^-5^ ± 0.929*10^-5 a^	1.49*10^-5^ ± 1.08*10^-5 a^	1.18*10^-5^ ± 0.560*10^-5 ab^
Sr	3.13*10^-5^ ± 1.68*10^-5b^	3.71*10^-5^ ± 1.75*10^-5b^	6.73*10^-5^ ± 2.53*10^-5 a^	3.48*10^-5^ ± 1.24*10^-5b^	6.34*10^-5^ ± 2.22*10^-5 a^
Cd	3.05*10^-6^ ± 7.45*10^-6 ab^	8.19*10^-6^ ± 20.0*10^-6 a^	5.41*10^-6^ ± 12.7*10^-6 ab^	1.55*10^-6^ ± 5.74*10^-6b^	2.74*10^-6^ ± 8.20*10^-6 ab^
Ba	2.19*10^-5^ ± 0.972*10^-5b^	2.21*10^-5^ ± 1.53*10^-5b^	3.84*10^-5^ ± 1.84*10^-5 a^	2.09*10^-5^ ± 0.914*10^-5b^	3.90*10^-5^ ± 1.62*10^-5 a^
Tl	1.74*10^-5^ ± 5.13*10^-5 a^	7.16*10^-5^ ± 18.8*10^-5 a^	5.19*10^-5^ ± 12.3*10^-5 a^	2.29*10^-5^ ± 7.79*10^-5 a^	3.01*10^-5^ ± 10.4*10^-5 a^
Pb	6.83*10^-5^ ± 3.48*10^-5 a^	9.42*10^-5^ ± 17.5*10^-5 a^	7.30*10^-5^ ± 8.27*10^-5 a^	5.78*10^-5^ ± 4.24*10^-5 a^	7.54*10^-5^ ± 6.61*10^-5 a^
Na	0.401 ± 0.0636^c^	0.413 ± 0.0581^c^	0.575 ± 0.109 ^a^	0.283 ± 0.0875 ^d^	0.486 ± 0.0740^b^
Mg	0.0316 ± 0.00522 ^a^	0.0202 ± 0.00220^c^	0.0131 ± 0.00236 ^d^	0.0283 ± 0.00464^b^	0.0139 ± 0.00461 ^d^
Ca	0.0191 ± 0.00855^c^	0.0236 ± 0.00512^b^	0.02691 ± 0.00614 ^a^	0.0110 ± 0.00591 ^d^	0.0160 ± 0.0103^c^
K	0.373 ± 0.0673^c^	0.406 ± 0.0426^b^	0.169 ± 0.0371 ^e^	0.453 ± 0.0678 ^a^	0.283 ± 0.0535 ^d^

Results are expressed as mean values ± standard deviation, n = 30. Values in the same line with different superscripts were significantly different (*P* < 0.05).

### Selection of key elements and dimension reduction for modelling

The SWDA and one-way ANOVA were separately used to select key elements for machine learning modelling. Results of the SWDA showed that regarding the absolute content of the elements used, B, Fe, Ni, Cu, Sr, Na, Mg, K, and Ca were selected as the key variables; In contrast, as the relative content of related measuring elements, eight elements were selected as the key elements, namely Fe, Ni, Cu, Zn, Sr, Na, Mg, and K. Results of the one-way ANOVA showed that all the tested elements except Li, Be, Ti, Co, As, Cd, Tl, and Pb were selected considering the absolute content; and in terms of the relative content, the measured elements other than Be, B, Ti, Co, Tl, and Pb were selected as the key variables for modeling.

**Fig. 1:**
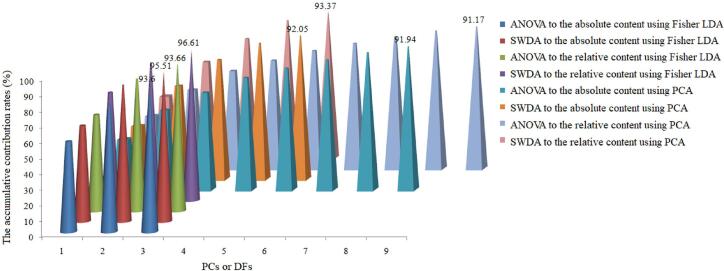
The accumulative contribution rates of the top several principal components (PCs) by principal component analysis (PCA) and discriminate functions (DFs) by Fisher linear discriminate analysis (Fisher LDA) for dimension reduction of the crucial input elements selected via stepwise discriminant analysis (SWDA) and one-way ANOVA.

Afterwards, PCA and Fisher LDA were utilized comparatively for dimension reduction of the key elements selected. The accumulative contribution rates of the top several principal components (PCs) and discriminate functions (DFs) used were shown in Fig. 1 It could be observed from Fig. 1 that the top 8 PCs and 5 PCs could be used to represent the key elements selected by using ANOVA and SWDA respectively on the absolute content dataset of the measured elements; as well as, the top 9 PCs and 5 PCs could represent the key elements selected via ANOVA and SWDA respectively on the relative content dataset of the measured elements. Fig. 1 also shows that the top 3 DFs could be used for representing these corresponding datasets respectively while SWDA was used.

**Fig. 2:**
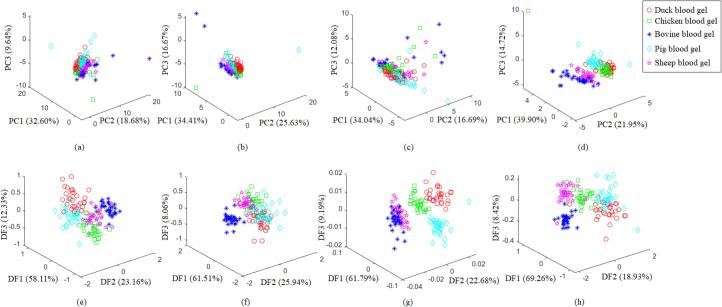
Scatter diagrams of the top 3 principal components (PCs) by principal component analysis (PCA) and the top 3 discriminate functions (DFs) by Fisher linear discriminate analysis (Fisher LDA) for dimension reduction of the crucial elements selected via stepwise discriminant analysis (SWDA) and one-way ANOVA. (a-ANOVA on the absolute content using PCA; b-SWDA on the absolute content using PCA; c-ANOVA on the relative content using PCA; d-SWDA on the relative content using PCA; e-ANOVA on the absolute content using Fisher LDA; f-SWDA on the absolute content using Fisher LDA; g-ANOVA on the relative content using Fisher LDA; h-SWDA on the relative content using Fisher LDA).

Fig. 2 shows that the scatter plots of the EABG samples under the top 3 PCs and 3 DFs separately. The figure could be used for exploring the distribution trends of the EABG samples with different species. The distinguishing capability of Fisher LDA was better than PCA. DFs of Fisher LDA calculated to fulfill the intra class deviation formed by each intra class projection value is as small as possible, and the inter-class deviation formed by the projection value between different classes is as large as possible (Han, Huang, Aheto, Zhang, & Feng, 2020); PCs of PCA was achieved by calculating the eigenvectors and eigenvalues of the covariance matrix of the original data matrix (Yousefi, Sfarra, Ibarra Castanedo, & Maldague, 2017). Even though PCA has been widely used for processing the multidimensional and serious collinearity datasets in a multivariate problem, Fisher LDA performed better than PCA for the task of classification herein.

### Results of ELM models

ELM models with different inputs obtained from section 3.2 were constructed and optimized for predicting the species of the EABG. During ELM modelling, one-third of the samples in each group were selected as the prediction set via the Kennard-Stone algorithm (Zhang et al., 2017). The rest samples were utilized as the training set.

According to the knowledge of ELM theory, the input weight and networks bias were generated randomly. Hence, each ELM model was performed 12 times for performance comparison. Table 3 shows the performances of the ELM models constructed for the testing samples. The table indicates that the optimal ELM models were obtained when the one-way ANOVA was used to select key elements and the Fisher LDA was used to reduce dimension. For consideration of the absolute content of the measured elements, while the Sig active function was used, the ELM model offered identification accuracy over 90%; as for the relative content considered, while the Sig and Sin functions were used, both ELM models offered high identification accuracies not lower than 93.0%.

**Table 3:** The identification accuracies of ELM models constructed for the unknown samples with different inputs, namely two original datasets including the absolute and relative content of the elements measured, two techniques for key elements selection including stepwise discriminant analysis (SWDA) and one-way ANOVA, two methods for dimension reduction including principal component analysis (PCA) and Fisher linear discriminate analysis (Fisher LDA), and three activation functions including Hardlim, Sig, and Sin.

Original dataset	Key elements selection	Dimension reduction	Activation function	Identification accuracy (%)
Absolute content	ANOVA	PCA	Hardlim	57.5 ± 2.72^j^
			Sig	58.5 ± 2.72^j^
			Sin	53.2 ± 2.37^k^
		Fisher LDA	Hardlim	89.7 ± 1.37^bc^
			Sig	91.5 ± 0.87^ab^
			Sin	90.8 ± 0.99^b^
	SWDA	PCA	Hardlim	70.7 ± 1.25^g^
			Sig	71.2 ± 2.37^g^
			Sin	69.7 ± 2.92^g^
		Fisher LDA	Hardlim	87.5 ± 1.19^cd^
			Sig	86.7 ± 1.25^cd^
			Sin	86.3 ± 1.37^d^
Relative content	ANOVA	PCA	Hardlim	64.5 ± 1.44^i^
			Sig	67.8 ± 3.10^h^
			Sin	57.0 ± 1.91^j^
		Fisher LDA	Hardlim	73.3 ± 5.19^f^
			Sig	93.0 ± 1.00^a^
			Sin	93.3 ± 1.25^a^
	SWDA	PCA	Hardlim	74.8 ± 2.23^f^
			Sig	77.5 ± 1.66^e^
			Sin	76.7 ± 2.62^e^
		Fisher LDA	Hardlim	90.3 ± 1.37^bc^
			Sig	88.67 ± 0.94^c^
			Sin	88.18 ± 1.28^c^

Results are expressed as mean values ± standard deviation, n = 12. Values in the same column with different superscripts were significantly different (*P* < 0.05).

Also, performances of the ELM models with original datasets from the absolute and relative content of the measured elements and the one-way ANOVA and SWDA were compared. Results showed that the performance of the ELM model using Fisher LDA for dimension reduction (88.3 ± 5.35%) was significantly superior to PCA (66.6 ± 8.31%) utilized; However, performance of the ELM model with original datasets from the absolute (76.1 ± 13.9%) and the relative content (78.8 ± 11.7%) of the measured elements showed no significant difference. As a comparison of the one-way ANOVA and SWDA used for variables selection, the key elements selected using SWDA were included in datasets selected via one-way ANOVA (see part 3.2), resulting in ANOVA datasets containing more specific information of the elements than AWDA datasets for EABG samples’ identification.

Eventually, for the 288 tests of ELM modelling, under the condition of using relative content and Sin active function, the best ELM model was obtained with the best neural network structure was 3–12-1, which provided the highest accuracy of 96.0% for the prediction set. It means that only two samples were misclassified of the unknown 50 samples. In training set, there were also only two samples misclassified offering a high prediction accuracy of 98.0%. It is suggest that elemental fingerprints accompanied by ELM have great potential in authenticating the edible animal blood foods.

## Conclusions

A method based on elemental fingerprint coupled with machine learning modelling was proposed for identifying the EABG species. Results suggest that: (1) both the absolute and relative content of the elements measured could be used for modelling; (2) Fisher LDA for dimension reduction was significantly better than PCA; (3) the optimal ELM models obtained with the relative content of the measured elements and Sin active function were used, which offered identification accuracies of not less than 96% in the training and test set. It can be concluded that the elemental fingerprint in conjunction with machine learning modeling has great potential in the species authentication of edible animal blood foods. This work presented the multi-element content in EABG for the first time, and developed a method for authenticating EABG species, which can be used to regulate the edible animal blood food market, thereby preventing illegal adulteration and unfair competition.

## Ethical Approval

The authors declare that this article does not contain any studies with human or animal subjects.

## Informed Consent

Not applicable, as this study does not include any human participants.

### CRediT authorship contribution statement

**Fangkai Han:** Funding acquisition, Conceptualization, Methodology, Data curation, Visualization, Writing – original draft. **Joshua H. Aheto:** Methodology, Writing – review & editing. **Marwan M.A. Rashed:** Investigation, Writing – review & editing. **Xingtao Zhang:** Investigation, Supervision.

## Declaration of Competing Interest

The authors declare that they have no known competing financial interests or personal relationships that could have appeared to influence the work reported in this paper.
